# Influence of Post-Treatment with 75% (v/v) Ethanol Vapor on the Properties of SF/P(LLA-CL) Nanofibrous Scaffolds

**DOI:** 10.3390/ijms13022036

**Published:** 2012-02-14

**Authors:** Kui-Hua Zhang, Qing Ye, Zhi-Yong Yan

**Affiliations:** College of Materials and Textile Engineering, Jiaxing University, Zhejiang 314001, China; E-Mails: yeqing198984@hotmail.com (Q.Y.); yzyong77@mail.zjxu.edu.cn (Z.-Y.Y.)

**Keywords:** electrospinning, SF/P(LLA-CL), post-treatment

## Abstract

In order to improve the water-resistant ability of silk fibroin (SF) and SF/P(LLA-CL) blended nanofibrous scaffolds for tissue engineering applications, 75% (v/v) ethanol vapor was used to post-treat electrospun nanofibers. SEM indicated that the treated SF and SF/P(LLA-CL) nanofibrous scaffolds maintained a nanofibrous structure and possessed good water-resistant ability. Characterization of ^13^C CP-MAS NMR clarified that 75% (v/v) ethanol vapor could induce SF conformation from random coil or α-helix to β-sheet. Although the water contact showed that treated SF/P(LLA-CL) blended nanofibrous scaffolds were hydrophobic, the water uptake demonstrated that their hydrophilicity was greatly superior to those of pure P(LLA-CL) nanofibrous scaffolds. Furthermore, the treated SF/P(LLA-CL) nanofibrous scaffolds, both in dry state and wet state, could retain good mechanical properties. Therefore, 75% (v/v) ethanol vapor treatment might be an ideal method to treat SF and SF/P(LLA-CL) nanofibrous scaffolds for biomedical applications.

## 1. Introduction

Natural extracellular matrix(ECM)is composed of a cross-linked porous network of nano-sized multifibril collagens embedded in glycosaminoglycans [1,2]. Electrospinning has been broadly recognized as a unique and facile technique for producing nanofibers, which could physically biomimic the structure of the extracellular matrix (ECM) of native tissues [3,4].

Silk fibroin (SF) is an attractive natural fibrous protein for tissue engineering applications such as skin, bone, cartilage, vascular blood and neve repair due to its unique properties including good biocompatibility and biodegradability, low inflammatory response and commercial availability at relatively low cost [5–8]. However, electrospun SF nanofibrous scaffolds possess weak mechanical properties. In tissue engineering, the electrospun scaffolds should physically resemble the nanofibrous features of extracellular matrix (ECM) with suitable mechanical properties for maintaining the stability of the scaffolds before the cells can produce their own ECM [9]. In order to improve mechanical properties, we have successfully fabricated electrospun SF/P(LLA-CL) blended nanofibrous scaffolds for tissue engineering application. All results demonstrated that they not only greatly promoted cells growth but also possessed good mechanical properties [10,11]. However, SF component of electrospun SF/P(LLA-CL) blended nanofibrous scaffolds without any modification was sensitive to water and thus could not keep its structure when used as tissue engineering scaffolds. Therefore, it is necessary to post-treat in order to enhance their ability of water resistance.

The structure of SF nanofibrous matrices was transformed from random coils or helix (silk I) to β-sheet (silk II) through the post-treatment methods of methanol and ethanol soaking, or their vapor and water vapor treatment [12–15]. To date, we found 75% (v/v) ethanol vapor treatment process not only induced silk fibroin conformation from random coils to β-sheet, but also was an effective sterilization approach to SF nanofibrous scaffolds [16]. Such a method is advantageous for nanofibrous matrices containing bioactive materials for tissue engineering applications, which could avoid bioactive materials loss to a large extent in post-treatment and sterilization process.

In the present study, electrospun SF and SF/P(LLA-CL) blended nanofibrous scaffolds with different weight ratios were treated with 75% (v/v) ethanol vapor. The properties of nanofibrous scaffolds, after being treated with 75% (v/v) ethanol vapor, such as morphologies, hydrophilicity and mechanical properties were evaluated.

## 2. Results and Discussion

### 2.1. Nanofibrous Morphologies Before and After Treatment

**Figure 1:**
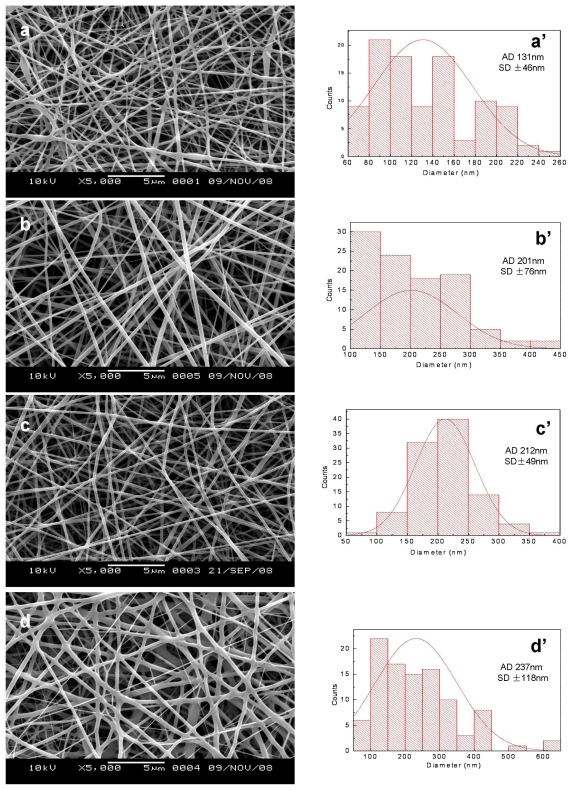
SEM images and diameter distributions of electrospun SF/P(LLA-CL) nanofibers with different ratios ((**a**, **a**′) 100:0; (**b**, **b**′) 75:25; (**c**, **c**′) 50:50; (**d**, **d**′) 25:75).

**Figure 2:**
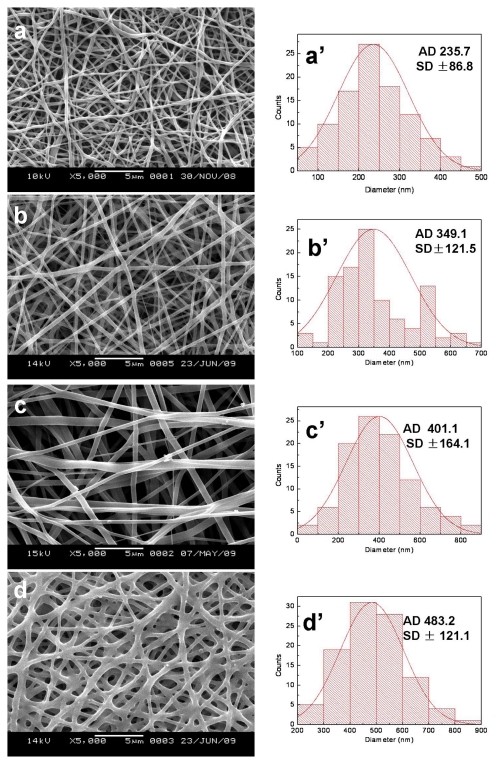
SEM images and diameter distributions of electrospun SF/P(LLA-CL) nanofibers with different weight ratios after being treated with 75% (v/v) ethanol vapor ((**a**, **a**′) 100:0; (**b**, **b**′) 75:25; (**c**, **c**′) 50:50; (**d**, **d**′) 25:75).

**Figure 3:**
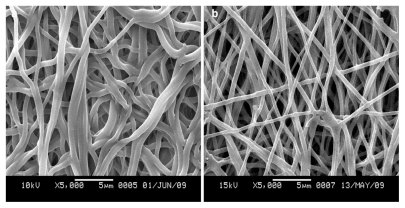
SEM images of electrospun SF/P(LLA-CL) (50:50) nanofibers after being soaked in water ((**a**) non-treated; (**b**) treated with 75% (v/v) ethanol vapor).

SEM micrographs and diameter distributions of electrospun SF/P(LLA-CL) nanofibrous scaffolds before and after being treated with 75% (v/v) ethanol vapor were shown in Figures 1 and 2. Compared with non-treated nanofibers, the morphologies of treated pure SF nanofibers and SF/(PLLA-CL) blended nanofibers (the weight ratio of 75:25 and 50:50) had no obvious difference. However, treated SF/ (PLLA-CL) blended nanofibers with a weight ratio of 25:75 experienced greater change and most of the nanofibers were connected to each other. The effect might be caused by the fact that P(LLA-CL), a good visco-clastic copolymer, was swollen by the ethanol vapor. The average diameters of treated nanofibers enhanced in comparison with non-treated relevant nanofibers. SEM micrographs of electrospun SF/P(LLA-CL) (50:50) nanofibrous scaffolds after soaking in deionized water for four days were shown in Figure 3. During the experiment, the non-treated nanofibrous scaffolds were found to shrink immediately after being soaked in deionized water. As shown in Figure 3a, the nanofibers were obviously swollen and bonded with each other. However, the treated nanofibrous scaffolds after being soaked in water still maintained good morphologies. The results indicated that 75% (v/v) ethanol vapor was effective to treat SF/P(LLA-CL) nanofibers. Thus, this method meets the needs to maintain nanofibrous structure to biomimic ECM after treatment

### 2.2. Structure Analysis of Nanofibrous Scaffolds Before and After Treatment

**Figure 4:**
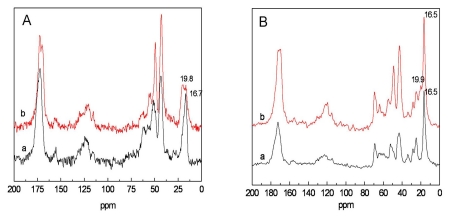
^13^C CP/MAS NMR spectra of electrospun and treated SF and SF/P(LLA-CL) (50:50) blended nanofibers with 75% (v/v) ethanol vapor. **A**, pure SF (a, non-treated; b, treated with 75% (v/v) ethanol vapor); **B**, SF/P(LLA-CL) (50:50) ((a) non-treated; (b) treated with 75% (v/v) ethanol vapor).

The secondary structure of *Bombyx mori* silk fibroin is composed of the major conformations including random coils or helix (silk I) and β-sheet (silk II) [17]. The β-sheet structure can be identified by the ^13^C chemical shifts of Gly (glycine), Ser (serine) and Ala (alanine) that are indicative of β-sheet conformations. Particularly, the chemical shift of alanyl C^β^ is an excellent indicator of the silk fibroin conformation [18,19]. The ^13^C NMR spectra for electrospun and treated SF and SF/P(LLA-CL) (50:50) blended nanofibers with 75% (v/v) ethanol vapor were shown in Figure 4. The chemical shift of Ala C^β^ in SF nanofibers varied from 16.7ppm for random coils or helix to 19.8 ppm for β-sheet conformation after treatment with 75% (v/v) ethanol vapor. The peak at 19.9 ppm was appeared in ^13^C NMR spectra of SF/P(LLA-CL) (50:50) blended nanofibers after treatment with 75% (v/v) ethanol vapor, which was the chemical shift of Ala C^β^ for β-sheet conformation. Furthermore, the peak at 172.0 ppm for carbonyl carbons split into a doublet after being treated with 75% (v/v) ethanol vapor. The split was due to conformation from random coil to β-sheet [19]. The results demonstrated that the conformation of SF converted from random coil to β-sheet after being treated with 75% (v/v) ethanol vapor.

### 2.3. The Hydrophilicity of Nanofibrous Scaffolds

The hydrophilic-hydrophobic property of nanofibrous scaffolds was evaluated by static water contact angle and water uptake. The former reflected transient hydrophilic-hydrophobic property of material surface, and the latter reflected hydrophilic-hydrophobic property of the material surface and internal body in a relatively long period. The water contact angle and water uptake of SF/P(LLA-CL) nanofibrous scaffolds with different weight ratios before and after being treated with 75% (v/v) ethanol vapor were shown in Table 1. Before treatment, pure SF nanofibrous scaffolds showed ultra-hydrophilicity because of its hydrophilic groups and random coils conformation. The pure P(LLA-CL) nanofibrous scaffolds showed an angle around 120°, indicating that P(LLA-CL) nanofibrous scaffolds were hydrophobic. With the increasing ratio of SF from 25 to 75 in the blended nanofibrous scaffolds, the water contact angles of the nanofibrous scaffolds decreased from 87.9° to 75.5°. The hydrophobility of P(LLA-CL) nanofibrous scaffolds could be transformed to hydrophilicity by introducing SF ingredient. After treatment, the water contact angle of pure SF nanofibrous scaffolds was 49.5° and still possessed good hydrophilicity. More interestingly, the results of the water contact angle showed that the blended nanofibrous scaffolds with the weight ratios from 75:25 to 25:75 transformed from hydrophilicity to hydrophobility, and the water contact angle of nanofibrous scaffolds with the weight ratio of 50:50 achieved maximum (130.5°), and then began to decrease. The possible reasons were listed as follows: (1) silk fibroin conformation transformed from random coil (soluble in water) to β-sheet (insoluble in water) after treatment; (2) The relative shrinkage of nanofibrous scaffolds after treatment led to the decrease of pore diameter and porosity; (3) silk fibroin of β-sheet structure possessed high crystallinity, but P(LLA-CL) was a kind of good elastomer. Therefore, as the blended ratio was 50:50, the surface of nanofibers could become rough, which resulted in greater water contact angle [20,21]. However, from the point of water uptake, the hydrophilicity of the blended nanofibous scaffolds after treatment had great superiority over pure P(LLA-CL) nanofibrous scaffolds. The reason may be that SF molecules contained multiple hydrophilic functional groups, such as amido (−NH_2_), carboxyl (−COOH) and hydroxyl (−OH). In addition, pure SF and SF/P(LLA-CL) blended nanofibrous scaffolds presented larger pore diameter and higher specific surface area in comparison with pure P(LLA-CL) nanofibrous scaffolds [10]. Such materials may be conducive to vascular regeneration, because there are weak interactions between hydrophobic surface with lower surface free energy and ingredients in blood, which could display excellent anticoagulant activity [22]. Meanwhile, over a relatively long period, the blended SF/P(LLA-CL) nanofibrous scaffolds possessed good hydrophilicity, which could be beneficial to cell adhesion, proliferation and migration.

### 2.4. Mechanical Properties of Nanofibrous Scaffolds Before and After Treatment

**Figure 5:**
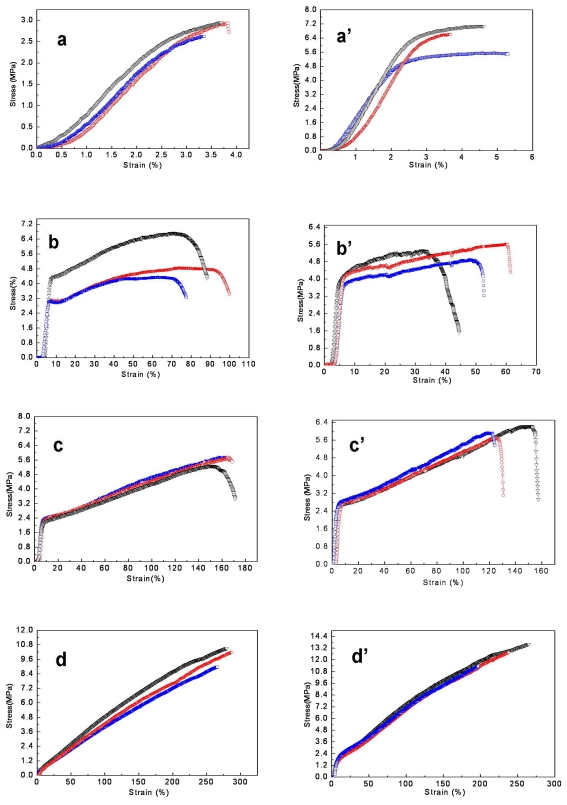
The stress-strain curves of SF/P(LLA-CL) nanofibrous scaffolds with different weight ratios before and after treatment with 75% (v/v) ethanol vapor. ((**a**, **a**′) 100:0; (**b**, **b**′) 75:25; (**c**, **c**′) 50:50; (**d**, **d**′) 25:75).

The typical tensile stress-strain curves of SF/P(LLA-CL) blended nanofibrous scaffolds with varied weight ratios before and after treatment with 75% (v/v) ethanol vapor were shown in Figure 5. The average elongation at break and the average tensile strength of each specimen were summarized in Table 2. Figure 5 and Table 2 showed that the pure SF nanofibrous scaffolds were typical brittle fracture and the average elongation at break was only 3.85% ± 0.30 and the average tensile strength was 2.72 ± 0.60 MPa. With increasing the blended ratio of P(LLA-CL) to SF in the range from 25 to 75, the nanofibrous scaffolds transformed from brittle to flexible and both the elongation at break and the average tensile strength increased obviously. Furthermore, the yield point appeared at the weight ratios of 75:25 and 50:50. After treatment, the average elongation at break and the average tensile strength of pure SF nanofibrous scaffolds increased slightly. In tensile test, we found that pure SF nanofibrous scaffolds ruptured easily at two terminals near fixture due to its friability to result in the error of measurement. For SF/P(LLA-CL) nanofibrous scaffolds with different weight ratios, the average elongation at break decreased to some extent but the average tensile strength had no obvious difference. This was mainly caused by the intermolecular and intramolecular covalent bonds and the physical entanglements formed among nanofibers, whereby the decrease of sliding in chains and among fibers led to decreasing the elongation at break.

**Figure 6:**
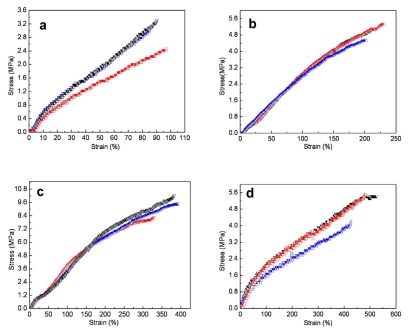
The stress-strain curves of SF/P(LLA-CL) nanofibrous scaffolds with different weight ratios in wet state. ((**a**) 75:25; (**b**) 50:50; (**c**) 25:75; (**d**) 100:0).

Mechanical tests of pure SF scaffolds in wet state cannot be carried out because the tenacity is too poor to be measured. In comparison with treated blended nanofibrous scaffolds in dry state, all average tensile strength decreased to some extent, whereas all the average elongation at break obviously increased in wet state, and yield point disappeared in stress–strain curves (Figure 6). In addition, compared with pure P(LLA-CL) nanofibrous scaffolds in dry state, the average tensile strength decreased and the average elongation at break increased in wet state to some extent. The plasticizing effect of water might be contributed to the tensile behavior of soaked SF/P(LLA-CL) blended nanofibrous scaffolds.

## 3. Experimental Section

### 3.1. Materials

Cocoons of *Bombyx mori* silkworm were kindly supplied by Jiaxing Silk Co. Ltd. (China). A copolymer of P(LLA-CL) (50:50), which has a composition of 50 mol% l-lactide, was used. 1,1,1,3,3,3,-hexafluoro-2-propanol (HFIP) was purchased from Daikin Industries Ltd. (Japan). Ethanol was obtained from Chemical Reagent Co., Ltd. (China)

### 3.2. Post-Treatment of SF/P(LLA-CL) Nanofibrous Scaffolds

Pure SF and SF/P(LLA-CL) nanofibrous scaffolds were fabricated according to reference [10] and then were treated with 75% (v/v) ethanol vapor to induce a β-sheet conformational transition, which resulted in insolubility in water. Briefly, 75% (v/v) ethalnol vapor-treated samples were prepared by placing SF and SF/P(LLA-CL) nanofibrous scaffolds in a desiccator saturated with 75% (v/v) ethanol vapor at 25 °C for 6 h and then dried in a vacuum at room temperature for 24 h.

### 3.3. Characterization

The morphology was observed with a scanning electronic microscope (SEM) (JSM-5600, Japan) at an accelerated voltage of 15 KV. The mean fiber diameters were estimated using an image analysis software (Image-J, National Institutes of Health, USA) and calculated by selecting 100 fibers randomly observed on the SEM images.

The ^13^C CP-MAS NMR spectra of the electrospun scaffolds were obtained on NMR spectrometer (Bruker AV400, Switzerland) with a ^13^C resonance frequency of 100 MHz, contact time of 1.0 ms, pulse delay time of 4.0 s.

### 3.4. Contact Angle Measurements and Water Uptake

Surface wettabilities of the electrospun scaffolds were characterized by the water contact angle measurement. The images of the droplet on the membrane were visualized through the image analyzer (OCA40, Datephysics Co, Germany) and the angles between the water droplet and the surface were measured. The measurement used distilled water as the reference liquid and was automatically dropped onto the electrospun scaffolds. To confirm the uniform distribution of blend nanofibrous scaffolds, the water contact angle was measured 3 times from different positions and an average value was calculated by statistical method.

The dry treated different scaffolds (50 mm × 50 mm) were immersed into distilled water for 24 h at room temperature. The wet scaffolds were immediately weighed after removing the surface water with a filter paper. The water uptake was calculated according to the following equation:

$$water\;uptake\;(\%)=\frac{w_{w}-w_{d}}{w_{d}}\times 100\%$$

Where *W_w_* and *W_d_* were the weight of wet and dry scaffolds, respectively.

### 3.5. Mechanical Property Measurements

Mechanical properties were obtained by applying tensile test loads to specimens prepared from the electrospun SF/P(LLA-CL) scaffolds. In this study, three specimens were prepared according to the method described by Huang *et al.* [23]. Mechanical properties were tested by a materials testing machine (H5K-S, Hounsfield, England) at the temperature of 20 °C, a relative humidity of 65% and an elongation speed of 10 mm/min. The specimen thicknesses were measured using a digital micrometer, having a precision of 1 μm. Wet state samples were also measured after soaking electrospun scaffolds in distilled water for 1 h and the water on the surface of scaffolds was blotted with filter paper. The specimen thicknesses were measured using a digital micrometer, having a precision of 0.01 mm.

## 4. Conclusions

In the present study, to improve the stability of SF/P(LLA-CL) nanofibrous scaffolds *in vitro* and *in vivo*, the conformation of SF in SF/P(LLA-CL) nanofibrous scaffolds was greatly altered from random coil or α-helix to β-sheet by treating samples with 75% (v/v) ethanol vapor. The results of SEM and water resistant test showed 75% (v/v) ethanol vapor was a predominant post-treatment method. Treated SF/P(LLA-CL) nanofibrous scaffolds still possessed excellent hydrophilicity and mechanical properties. Such a method might be beneficial to tissue engineering scaffolds and as a vehicle for drugs.

## Figures and Tables

**Table 1:** Water contact angle and water uptake of SF/P(LLA-CL) nanofibrous scaffolds with various blended ratios. Data are representatives of three independent experiments and all data are used as means ± SD.

Sample		SF:P(LLA-CL) weight ratios				
	
		100:0	75:25	50:50	25:75	0:100
Water contact	Untreated	——	75.5 ± 1.2	78.4 ± 2.0	87.9 ± 2.7	120 ± 3.2
angle (°)	Treated	49.5 ± 2.8	121.9 ± 1.5	130.5 ± 0.9	121.8 ± 1.9	——
Water uptake (%)	Treated	353.67 ± 3.45.45	259.79 ± 3.21	200.67 ± 4.014.01	130.74 ± 2.092.09	10.92 ± 2.762.76

**Table 2:** Mechanical properties of SF/P(LLA-CL) blended nanofibrous scaffolds with various blended ratios before and after treatment with 75% (v/v) ethanol vapor as well as in wet state. Data are representatives of three independent experiments and all data are used as means ± SD.

SF/P(LLA-CL) weight ratio			Average specimen thickness (mm)	Average elongation at break (%)	Average tensile strength (MPa)
100:0	Dry	Untreated	0.050 ± 0.005	3.85 ± 0.30	2.72 ± 0.60
		Treated	0.054 ± 0.005	4.50 ± 0.80	6.39 ± 0.78
	Wet		—	—	—
75:25	Dry	Untreated	0.082 ± 0.006	98.86 ± 16.98	5.28 ± 1.25
		Treated	0.055 ± 0.006	47.95 ± 13.07	5.28 ± 0.37
	Wet		0.070 ± 0.002	90.93 ± 5.89	2.25 ± 0.30
50:50	Dry	Untreated	0.075 ± 0.004	168.75 ± 29.70	5.62 ± 1.61
		Treated	0.078 ± 0.002	132.90 ± 16.03	5.96 ± 0.29
	Wet		0.088 ± 0.003	215.75 ± 14.60	4.98 ± 0.42
25:75	Dry	Untreated	0.078 ± 0.008	279.67 ± 34.98	10.60 ± 2.45
		Treated	0.082 ± 0.005	232.79 ± 33.99	12.59 ± 1.11
	Wet		0.072 ± 0.003	369.16 ± 33.57	9.32 ± 1.03
0:100	Dry	Untreated	0.088 ± 0.005	458.83 ± 19.35	6.29 ± 1.30
		Treated	—	—	—
	Wet		0.068 ± 0.004	479.11 ± 50.18	5.04 ± 0.72
